# Recovery of Mineral Oil from Underground Electrical Cables

**DOI:** 10.3390/ijerph16132357

**Published:** 2019-07-03

**Authors:** Stefano Caimi, Claudio Colombo, Raffaele Ferrari, Giuseppe Storti, Massimo Morbidelli

**Affiliations:** Institute for Chemical and Bioengineering, Department of Chemistry and Applied Bioscience, ETH Zurich, 8093 Zurich, Switzerland

**Keywords:** oil recovery, environmental threat, oil-filled cables, electrical cables, underground cables

## Abstract

To remove the mineral oil impregnating the insulating paper present in old, disconnected, underground electrical cables, which represents a threat to the environment, two approaches are investigated at laboratory (1 m) and pilot (10 m) scales. The first one involves in situ polymerization to clog the inner channel of the cables and to enable the washing of the outer paper region impregnated by the oil by axial flow of a displacing fluid (water). The second approach leaves the inner channel open and employs repeated cycles of pressurization and rest to displace the oil contained in the paper by radially pushing the water from the inner channel into the outer layers. The pressurization and rest times were optimized to obtain the highest oil extraction rate. While the first approach showed limitations in terms of required pressures and operating time, which increase with the length of the cables, the second one was effective at removing 97% of the oil impregnating the paper layers within 25 cycles. Even more relevant, this second solution, in contrast to the first one, can be easily scaled up as it does not depend on the length of the cable, and was successfully tested on a 10 m cable, showing 98% oil recovery.

## 1. Introduction

Oil-filled electrical cables have been placed underground all over Europe since the late 1950s to distribute electric power throughout the territory, particularly in urban areas. After many years of operation, these cables are disconnected and abandoned, thus posing a logistic and environmental problem. Indeed, the insulating mineral or synthetic oil contained in the cables may accidentally leak out as a consequence of material deterioration, ground movements, failure at jointures between cables, and damage during drilling/excavations. The released oil can percolate through the ground and eventually reach groundwater reservoirs, thus raising significant concerns with respect to the public health and the environment in general [1]. It is therefore necessary to either remove the cables by excavation or empty them of the insulation oil. Despite the first strategy guaranteeing a complete recovery of the oil as well as of the other materials composing the cables, this is seldom applicable due to the inaccessibility of the cables as additional infrastructures may have been built on them, especially in highly populated urban areas. Moreover, when feasible, this method would result in high costs due to the manpower needed for the excavations and expensive drilling machinery, as well as long time and interference with the city traffic. Therefore, an in situ method for the oil recovery not requiring cable removal from the ground is highly desirable.

**Figure 1:**
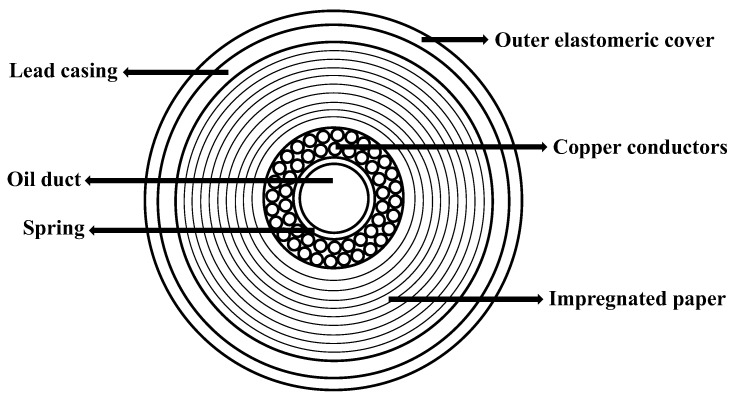
Components of a typical high-voltage electrical cable.

As sketched in Figure 1, conventional cables are made of an inner oil-filled channel surrounded by the copper conductors. The latter are held together by a metallic spring and are surrounded by a multitude of layers of high-density paper which guarantee electric insulation, low dielectric losses, and high oil permeability [2]. While the oil present in the inner channel of the cable can be easily removed by a displacement fluid (e.g., water), a significant part of it remains, impregnating the paper layers closely packed around the conductors. 

Different approaches have been proposed aimed to clean electrical cables from the impregnating oil. The use of microorganisms to be injected in the cable and capable of digesting the oil (bioremediation) has been intensively studied, but open issues such as the choice of effective microorganisms, the need for nutrient supply, and the lack of oxygen still remain to be solved [3,4,5]. Another possible in situ approach is the chemical oxidation of the oil using different reagents [6,7,8,9,10]. However, this method is far from being applicable as it entails the use of chemicals and it involves a safety risk associated with the production of gases in a closed environment along with the possible occurrence of nonspecific reactions. More promising approaches have been proposed based on the displacement of the oil by suitable sequestering solutions, typically aqueous solutions of emulsifiers able to disperse the oil in the form of small droplets. Eventually, the collected emulsion has to be properly destabilized in order to separate the oil from the aqueous phase, thus requiring an additional, nontrivial separation step in order to recycle the displacement agent [11]. The use of hydrogels with larger viscosity has been reported as providing larger displacing power [12]. However, this approach could be even more problematic with respect to the following separation step. Therefore, an effective and easily applicable method for in situ removal of the oil from underground cables is still missing.

In this work, two different methods have been developed and investigated. The first one consists in fully clogging the inner channel of the cable by in situ polymerization followed by water injection into the paper annular region to remove the impregnating oil by displacement in the axial direction. The second approach leaves the inner channel open but applies repeated cycles of pressurization and rest to radially push the water from the channel into the paper layers and displace the oil, which accumulates in the inner channel during the cycle at low pressure. The two methods have been developed and the feasibility of the most effective one has been assessed both at the laboratory and at the pilot scale.

## 2. Materials and Methods

### 2.1. Materials

The following chemicals have been employed without further treatments: butyl acrylate (BA, purity >99%) and methyl methacrylate (MMA, purity >99%) from Sigma Aldrich; Benzoyl peroxide (BP, purity >75%) from Aldrich-Fine Chemicals.

As shown in Figure 1, the cable exhibits a multilayer structure with the central, open duct where the oil freely flows. Around the duct, two layers of multiple copper conductors are kept in position by a metallic spring. The wires are surrounded by hundreds of layers of superimposed insulation paper, impregnated by oil. The outer layers of the cable are made of a lead casing covered by an elastomeric sheath. The diameter of the inner channel is 14 mm and the thickness of the paper layers is 17 mm.

### 2.2. Methods

**Approach 1**—The in situ polymerization was conducted by filling the cable with a mixture of monomers (MMA/BA 50/50 wt.%) together with a suitable thermal initiator (azobisisobutyronitrile, AIBN, 5 wt%). The system was heated up using a heating cable to 70 °C for 6 hours to ensure reaction ignition and full conversion of the monomers. Once the inner channel was clogged, water was injected into the paper region using a volumetric piston pump (Ismatec ISM321 FMI007, Wertheim, Germany) and an injection cap designed to distribute the inlet water flow into the paper region. The system (closed at the opposite end) was initially pressurized to 6 bar and then left at rest till the pressure reduced below 1 bar. Then, the free end of the cable was opened and the oil was collected by gravity. In order to maximize oil removal, automated repeated cycles of initial pressurization to 6 bar were performed (500 cycles at an average pressure of 3.4 bar) until the waterfront reached the cable’s end. As soon as the pressure reduced below 0.5 bar, the pump started to pressurize the system again to 6 bar. These experiments were carried out using a setup made of the following components:

- a balance to measure the mass of injected water
- a volumetric piston pump
- a manometer to measure the inlet pressure of the cable
- an injection cap designed to maximize the flow into the paper region
- a collecting cap
- a heating cable with a suitable temperature controller to heat up the inner channel and initiate the polymerization

**Approach 2**—In this case, the cable was pressurized by pumping pure water into the central open channel (opposite cable end was closed), leaving the system at constant pressure of at least 3 bar for a defined period of time, releasing the pressure by opening the valves at the cable ends, and leaving the cable at atmospheric pressure for a given period of time to allow the oil to radially percolate through the paper layers into the inner channel, where it could accumulate. This oil was then removed by flushing the channel in the axial direction with water and the procedure repeated until no more oil was extracted. To enable the pressure control of the cable, two pressure controlling valves were installed at the inlet and the outlet of the cable, respectively. The final setup was made of a balance to measure the amount of injected water, a volumetric piston pump (Ismatec ISM321 FMI007), a manometer to measure the pressure inside the cable, and the two valves mentioned above.

The operating procedure consisted of the following steps:

- Pumping tap water and pressurizing the system (in our case, at about 3 bar)
- Leaving the system under pressure for a specific time
- Opening the valves to reduce the pressure to atmospheric and remove the free water from the inner channel (e.g., by air injection or draining)
- Leaving the system empty for a specific time to allow the oil to exit the paper layers and accumulate in the inner channel
- Washing the inner channel with water to remove the accumulated oil
- Repeating the procedure until no more oil is extracted

Given the absence of any stabilizer (no emulsifiers), the collected water–oil emulsion was easily separated by decantation in 24 h. Then, the amount of extracted oil was quantified and the water reused for another cleaning cycle.

**Swelling measurements procedure**—In order to evaluate the variation of porosity inside the paper region due to water absorption, direct experiments of mass uptake have been performed. Using paper recovered from the original cables, the following procedure has been used:

- Intensive vacuum drying of the paper to remove any residual water or oil
- Paper weighing and soaking in the selected medium for 24 h
- Paper extraction from the solution and gentle removal of the medium excess by cleaning paper
- Final weighing to measure the mass increase due to swelling

**Figure 2:**
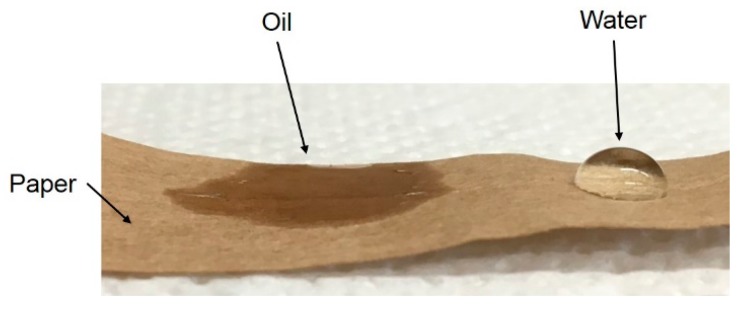
Wettability of a paper layer by oil and water after 1 min.

**Droplet sorption rate and contact angle measurements**—To evaluate the affinity between oil/water and paper, droplets of both pure liquids have been softly deposited onto dry paper and the behavior of the droplets followed in time. To prevent water evaporation (oil volatility at room temperature is negligible), these experiments were carried out inside a closed vessel containing water-saturated air. As an example, the picture in Figure 2 corresponds to about 1 minute after the droplet deposition.

While after a short time the oil appears to be quickly absorbed by the paper, the water droplet is still retaining its shape, thus indicating a much larger value of the contact angle. On the other hand, after 10 min, the water starts to wet and to be absorbed by the paper. After 30 min, the water droplet is no longer present and a wet darker region is clearly visible on the paper.

## 3. Results and Discussion

### 3.1. Approach 1—Axial Water Injection after Clogging the Inner Channel

**Figure 3:**
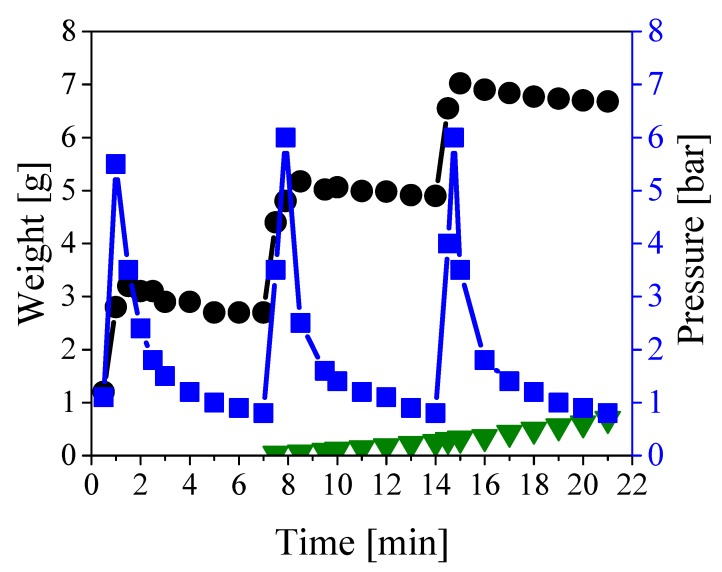
Weight of fed (black circles) and collected (green triangles) liquids, and pressure profile (blue squares) during the first three cycles of the pressurization/depressurization procedure.

As mentioned above, the oil present in the central duct can be easily removed by water pumping. This flow does not remove the oil impregnating the paper layers. To enable the recovery of such oil, the inner channel has to be carefully clogged so that water is forced to flow only through the paper region, thus displacing the oil. In order to polymerize the inner channel, the monomer and initiator mixture was pumped inside the central duct of the cable. Approximately 180 grams per meter of cable were needed to fill the inner channel and the open volume between the copper conductors. The cable was then heated with the heating cable for 6 hours to ensure full conversion of the monomers. Note that the implementation of this strategy in underground cables can be performed taking advantage of the internal copper conductors and exploiting their electrical resistance to heat up the system. The polymerization was repeated twice to clog any residual porosity resulting from the shrinking of the polymer during the reaction. Once the polymerization was complete, the cable inlet was sealed to the injection cap above-mentioned and the outlet to a collecting cap equipped with a valve to open/close the cable. The system was then subjected to repeated cycles of pressurization followed by rest to allow the displacing of the oil and its collection at the end of the cable. This pressurizing strategy was applied because the available pump was not able to ensure a constant pressure below the maximum pressure sustainable by the cable (about 6 bar). Therefore, to ensure a kind of constant flow at a flowrate much smaller than the minimum value provided by the available device, the pump was continuously switched on and off to prevent overpressure inside the cable. In order to extract as much oil as possible, the procedure was repeated for 500 cycles. The amounts of water fed and the oil collected as well as the pressure profile are shown in Figure 3 as a function of time for the first 3 pressurization/depressurization cycles.

As indicated by the blue squares in Figure 3, the system was pressurized to 6 bar approximately every 7 min, followed by a fast depressurization leading to a time-average pressure of almost 2 bar. The decrease in pressure reflects the oil displacement towards the open end of the channel, as the inlet cap was firmly closed and no leakage of water was observed. It is also worth noting that the pressure increased sharply as it is typical for noncompressible fluids. Indeed, about 2.6 grams of water were needed to fully pressurize 1 meter of cable, as shown by the black circles in Figure 3. The oil started being collected at the outlet of the cable after approximately 7 minutes, as shown in the same figure (green triangles), and this delay was due to the fact that the oil needed to fill all dead volumes at the cable outlet before appearing at the collecting port. By repeating the procedure for 500 cycles, about 550 grams of pure oil (water-free) were collected before the breakthrough of the displacing water. No more oil was displaced after such breakthrough, thus suggesting complete oil removal from the paper region. In order to evaluate the actual extent of oil removal, and at the same time check the extent of clogging of the inner channel achieved by polymerization, the cable was inspected by cutting it in both the axial and radial directions. 

**Figure 4:**
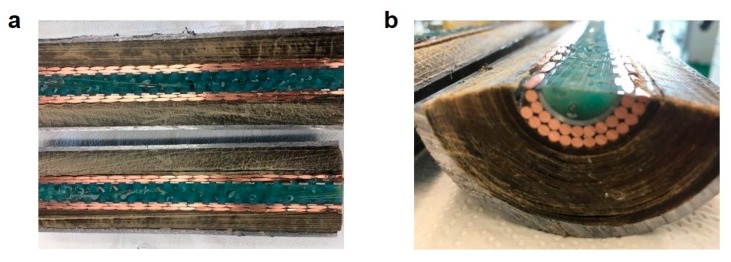
(**a**) Axial section and (**b**) cross-section of the cable after clogging by in situ polymerization and complete oil removal by water displacement.

As shown in Figure 4, the polymerization proved very effective in uniformly and completely clogging the inner channel and the free space among the copper conductors. Such uniform filling is the key requirement in order to prevent water channeling through residual free space and local bypass of the paper region. Moreover, the paper region was so dry that the removal of individual layers of dry paper was possible even by hand, thus confirming the complete oil removal.

**Figure 5:**
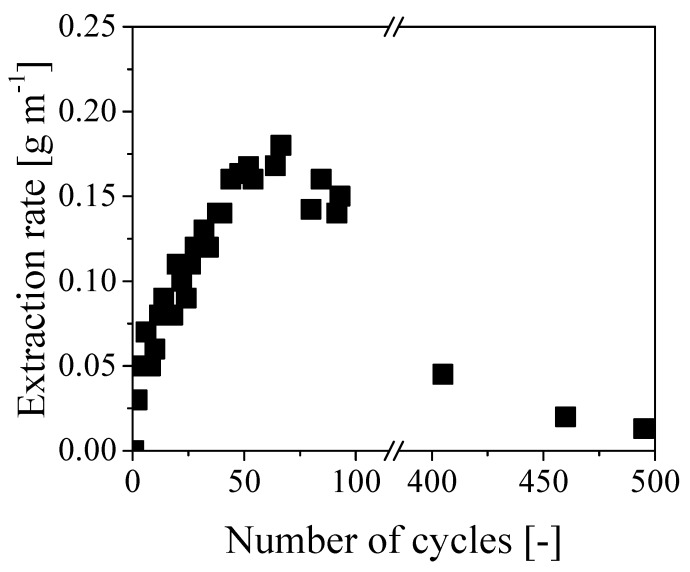
Evolution of the extraction rate with respect to the number of cycles.

Figure 5 reports the oil extraction rate as a function of the number of operating cycles. Notably, the extraction rate increased almost linearly for about 60 cycles up to a maximum value of 0.18 g min^−1^. As each cycle lasted about 7 minutes, this resulted in a maximum extraction rate of approximately 1.3 g cycles^−1^. On the other hand, the same rate started decreasing afterward, reaching values of 0.05 and 0.02 g min^−1^ after 400 and 500 cycles, respectively. Since the applied average pressure was quite constant all along the experiment, the initial increase of removal rate is consistent with the classical Darcy law [13]:

(1) $$Q=-A\frac{\kappa }{\mu }\frac{\Delta P}{L}$$

where *A* is the cross-section available to the flow, *μ* the medium viscosity, *κ* the overall permeability, *∆P* the pressure drop, and *L* the characteristic length for the flow (i.e., the cable length). Since during the experiment oil was replaced by water, viscosity decreased and an increasing flow rate was established. However, to explain the second stage with decreasing rate behavior after about 60 cycles, a decrease of permeability has to be considered, most probably due to a reduction of void fraction. This behavior was imputed to the paper swelling by water, resulting in reduced open cross-section available to the flow and, therefore, for a constant pressure, a decrease in flow rate. To quantify such an effect, swelling measurements were carried out on clean paper layers by soaking them in different media. The procedure is reported in the experimental section. The results of these measurements are summarized in Table 1 as percentage of mass swelling, defined as:

(2) $$\Phi =\frac{m-m_{0}}{m_{0}}100$$

where *m* and *m_0_* are the final and initial weight of the sample, respectively.

These results clearly show that the paper was swollen by water to a larger extent than oil. Accordingly, water swelling and the corresponding reduction of the cross-section available to water flow can be considered as the reasons for the decreasing rate of oil removal after the initial increase. Note that such decrease is overcoming the effect of the decreasing viscosity at increasing extent of oil removal already after about 60 cycles and becomes dominant at longer times. Equation (1) has been also used to estimate the initial value of the overall permeability of the cable, which was about 7.4 × 10^−10^ cm^2^ at a flow rate of 0.1 g min^−1^ and viscosity of 40 cP. This value has the same order of magnitude of permeability measured for oil reservoir rocks in semipervious environments [14] and it is definitely too low to enable an effective removal of oil in long tubes through axial displacement. More specifically, extremely large pressure values would be required in long cables to ensure a sustainable extraction rate: since the maximum pressure for these cables is about 6 bar, the time required to fully recover all the internal oil in a 1 km cable by water displacement, with an extraction rate of 0.1 g min^−1^, would be approximately 10 years. Thus concluding, even though the clogging of the inner channel by in situ polymerization was very effective and the oil was effectively displaced from the paper region by pure water, this procedure has no practical value because of the extremely small values of oil removal rates, which become even lower when increasing the cable length.

### 3.2. Approach 2—Radial Water Injection without Clogging the Inner Channel

This approach is based on a completely different strategy: instead of clogging the inner channel to prevent the free water flow into it and confine the water axial flow through the paper region at low permeability, the inner channel is directly filled with water and the entire cable is pressurized. Then, the system is left at high pressure for a given time (*t_press_*) while waiting for water radial penetration into the paper region by infusion under stagnant conditions. After such period of time, the pressure is quickly reduced to ambient pressure and the cable is left open and at rest for another given time (*t_rest_*). During this second stage, oil is draining from the paper region into the inner channel all along the cable axis and it can be collected right before the following pressurization step. The setup and the full operating procedure to be applied in this case are described in the experimental section. 

**Figure 6:**
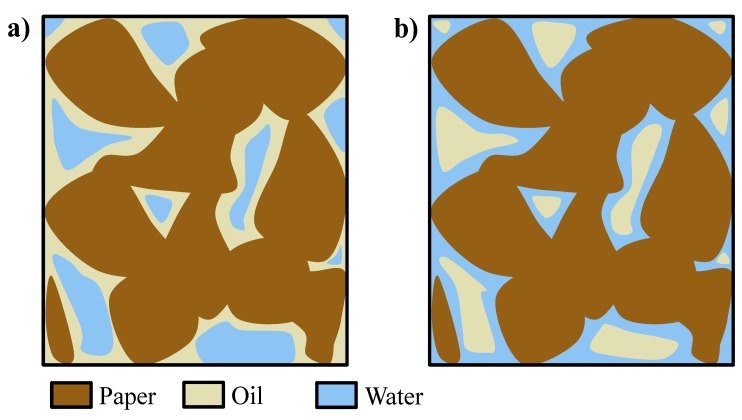
(**a**) Domains of water in oil at *t*_0_ and (**b**) domains of oil in water at *t_f_* [15,16].

The mechanistic concept underlying this second approach can be explained in terms of compatibility between paper and water or oil. As mentioned above in the context of Table 1, water sorption in the paper is larger than oil sorption, thus confirming its hydrophilic nature. To further verify this larger affinity and estimate the corresponding sorption kinetics, measurements of droplet sorption rate have been carried out as described in the experimental section. While the oil was promptly absorbed by the paper, the water droplet maintained a spherical shape for longer time, thus indicating hindered wettability and relevant hydrophobicity of the paper layer. It is worth noting that the paper used in these experiments was recovered from a real cable, and therefore it has been in contact with oil for a very long time. On the other hand, if a long enough contact time is applied, water is finally absorbed and, given its larger affinity for the paper, displaces the impregnating oil. Accordingly, the system goes through two different stages during the pressurization and resting steps of this procedure:

During the pressurization step, water penetrates into the paper region at the beginning of the phase (without wetting the paper) and remains entrained within the oil (which wets the paper), and water droplets or domains, surrounded by oil as continuous medium and not in contact with the paper, are formed (Figure 6a). However, at longer times (*t_f_*), water is slowly wetting the paper which has higher affinity with respect to it, thus displacing the oil and reversing the picture, finally forming oil droplets or domains inside an aqueous matrix in contact with the paper (Figure 6b) [15,16]. To verify this mechanism, a piece of paper impregnated by oil was soaked in water and left at rest; after some time, the oil was displaced and formed a layer at the top of the vessel, thus proving the displacing ability of the water with respect to the oil as well as the inversion of wetting fluid, from oil to water.

During the rest period, the dispersed oil phase is pushed out of the paper region towards the low-pressure inner channel, and drops into it during the rest time. After pressure equilibration, the final washing step is required to displace this recovered oil out of the cable before starting a new pressurization cycle.

**Figure 7:**
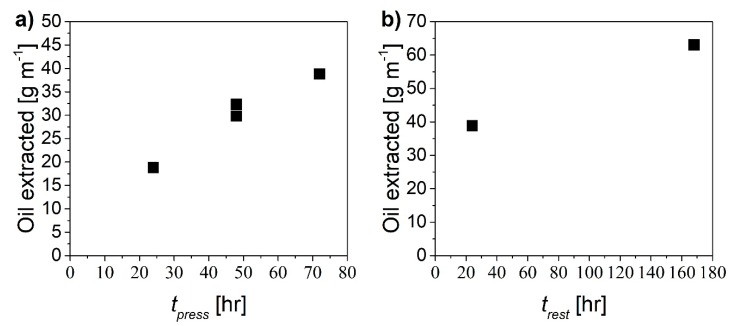
Amount of oil extracted as a function of (**a**) pressurization time at constant *t_rest_* equal to 24 h and (**b**) rest time at constant *t_press_* equal to 72 h.

The proposed mechanism should be effective to remove the oil by radial displacement through the paper region using pure water, provided that sufficient time for the two steps is allowed. In order to assess optimal values of pressurization time, *t_press_*, and rest time, *t_rest_*, several experiments were performed considering one single cycle. In particular, both times were varied from 24 to 72 hours and the amount of extracted oil was measured in each case. Figure 7 shows the amount of oil extracted as a function of the pressurization time (Figure 7a) and of the resting time (Figure 7b).

**Figure 8:**
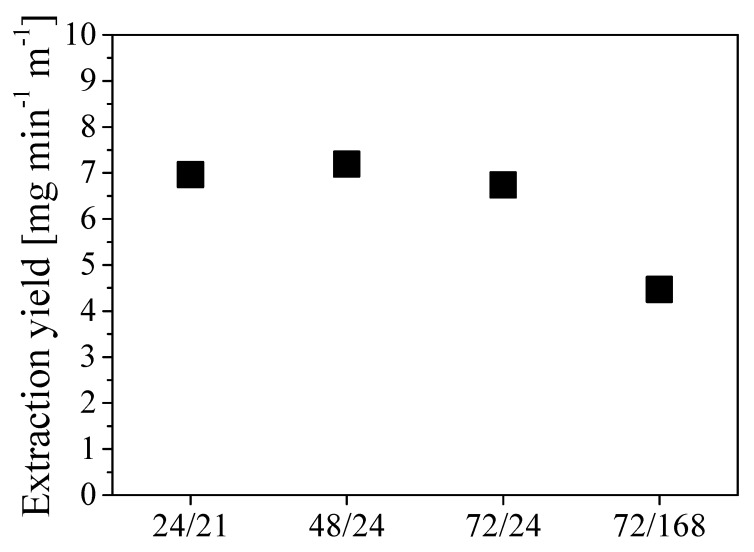
Extraction yield at different combinations *t_press_*/*t_rest_*, expressed in operating hours.

The larger the pressurization/rest time, the larger the extent of oil recovery. In order to comparatively evaluate the “productivity” of the different cases in terms of recovered oil, the following extraction yield Y[mg/(min·m)] was defined:

(3) $$Y=\;\frac{m_{oil}}{L\left(t_{press}+t_{rest}\right)}$$

where *m_oil_* is the mass of oil extracted and *L* is the tube length. The values of extraction yields estimated in the different cases are shown in Figure 8 as a function of different combinations of pressurization and rest times expressed in hours.

The two scenarios *t_press_*/*t_rest_* 24/21 and 48/24 exhibit the highest extraction yield, which resulted in being approximately 7 mg min^-1^ m^-1^. Given the low sensitivity of the extraction yield to the specific duration of each step for cycle time shorter than 3 to 4 days, the procedure *t_press_*/*t_rest_* 24/24 was selected for further investigations. This implies a cycle duration of about 3000 min with an oil removal of about 21 g cycle^−1^. 

**Figure 9:**
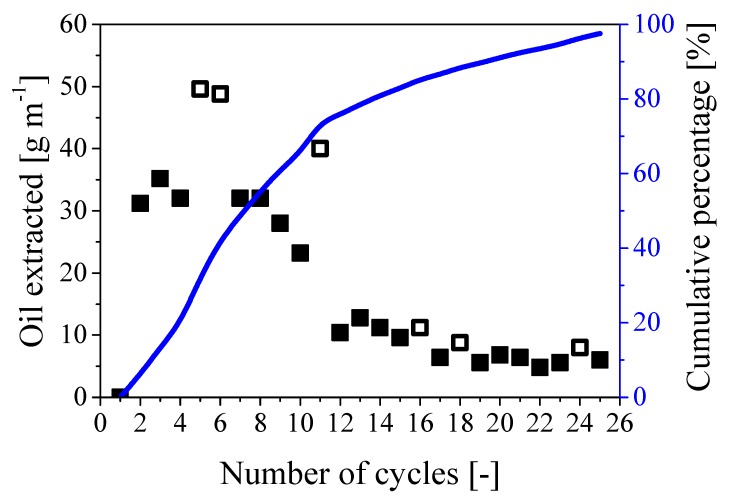
Amount of oil extracted during 25 repeated cycles. Symbols: mass of oil extracted per cycle; full symbols: 24/24; empty symbols: 24/48. Curve: cumulative percentage of extracted oil referred to the expected overall oil mass of 550 g m^−1^.

A series of cycles was implemented considering *t_press_*/*t_rest_* 24/24 using a cable 1m long. As the estimated oil content was about 550 g m^−1^, about 30 cycles were expected to be needed to fully remove the oil from the paper region, which corresponded to 2 months of treatment. The procedure was then repeated for 25 cycles and the amount of oil extracted per cycle is shown in Figure 9 together with the cumulative percentage referred to the expected value of 550 g of oil per meter of cable. 

As shown by the full symbols in the figure, the amount of extracted oil per cycle decreased from about 32 g cycle^−1^ m^−1^ during the first 8 cycles to about 7 g cycle^−1^ m^−1^ towards the end of the extraction process. This behavior is consistent with the proposed mechanism: while the water needs to penetrate a short radial distance into the paper region to displace the oil during the first cycles, larger radial distance to travel and paper swelling reduce the extraction rate. Nonetheless, 97% of the oil initially present in the cable was extracted in about 25 cycles (which corresponded to an operating time of 2 months), as shown by the curve representing the cumulative percentage in Figure 9. Note that the behavior remains practically the same in the case of larger rest time (24/48), as indicated by the empty symbols in the same figure.

Finally, as a first field test, the same procedure was applied to remove oil from a real 10 m long old cable, using the 24/24 cycle and the same pressure applied at the laboratory scale. About 5.4 kg of pure oil were collected in 20 cycles (overall recovery 98% with respect to the nominal oil mass), thus showing excellent reproducibility and scalability of the developed technology.

## 4. Conclusions

To address the environmental concerns related to the presence of toxic mineral oil in abandoned electrical cable, two different approaches to remove it were investigated. The first method involves the clogging of the inner channel of the cable by in situ polymerization, followed by axial water injection into the paper annular region to remove the impregnating oil by displacement. Despite this approach being effective and a complete recovery of oil from a 1 m cable being obtained, the pressure drop which is proportional to the length of the cable and the decreasing rate of oil removal in time make the scaling up unfeasible. The second approach involves repeated cycles of pressurization and rest to radially push the water from the inner unclogged channel into the paper region and displace the oil to the center of the cable from which it can be easily removed before the next pressurization step. This method proved to be effective and a recovery of 97% was obtained in 25 cycles which correspond to an operating time of approximately 2 months. The pressurization (24 h) and rest (24 h) times were optimized to have the highest oil extraction rate. Note that, in contrast to the first considered approach, this strategy is readily scalable and the optimal process times assessed at the laboratory scale (cable length of 1 m) apply to whatever the length of the cable under examination is, since the extraction process occurs in the radial rather than in the axial direction. Indeed, the same procedure was tested on a 10 m cable, showing a recovery of 98% of oil.

## Figures and Tables

**Table 1:** Percentage mass swelling of paper in different media.

Swelling Medium	Swelling Percentage, *Φ*
Water	60%
Oil	45%
